# A large outbreak of cryptosporidiosis at a pool in Melbourne, Australia, 2025: rapid investigation and public health response

**DOI:** 10.5365/wpsar.2026.17.2.1343

**Published:** 2026-06-30

**Authors:** Clarissa Moreira, Renae Oliver, Norelle L Sherry, Karolina Mercoulia, Annaliese van Diemen, Claire L Gordon

**Affiliations:** a North Eastern Public Health Unit, Austin Health, Heidelberg, Victoria, Australia.; b Microbiological Diagnostic Unit Public Health Laboratory, Department of Microbiology and Immunology at The Peter Doherty Institute for Infection and Immunity, The University of Melbourne, Melbourne, Victoria, Australia.; c Department of Infectious Diseases, The Peter Doherty Institute for Infection and Immunity, The University of Melbourne, Melbourne, Victoria, Australia.; d Department of Infectious Diseases and Immunology, Austin Health, Heidelberg, Victoria, Australia.

## Abstract

**Objective:**

Cryptosporidiosis is a gastrointestinal illness spread via infected people, animals, or contaminated water or food. This article describes a cryptosporidiosis outbreak associated with a swimming pool in Melbourne, Australia in February 2025.

**Methods:**

On 13 February, the North Eastern Public Health Unit was simultaneously notified of a reportt gastroenteritis symptoms by a social group who swam at Pool A on 4 February and of gastroenteritis symptoms among students at School B who attended the same pool on 5 February. An outbreak was declared and an investigation commenced. Communications were sent to 1034 pool patrons and schools that attended the pool in early February to advise of the outbreak, provide safe swimming messages and encourage testing among symptomatic individuals. Interviews with symptomatic individuals were undertaken. An online questionnaire supported active case finding and information-sharing. Local clinicians and laboratories were alerted to the outbreak and healthy swimming messages were promoted at the pool.

**Results:**

Cryptosporidiosis was suspected due to the 4–9-day incubation period. Due to initial reports of a high attack rate among the students and concerns about ongoing exposure to current pool users, the pool was immediately closed for hyperchlorination. Subsequent urgent faecal specimen testing confirmed *Cryptosporidium*. Overall, 16 confirmed and 59 probable cases were identified. The most common symptoms were diarrhoea (56%), abdominal pain (48%) and nausea (45%). Of those who only attended the pool once, the median incubation period was 7 days (range: 4–9 days). No cases were identified after hyperchlorination.

**Discussion:**

This pool-associated cryptosporidiosis outbreak demonstrates the importance of rapid outbreak investigation and response, and pre-emptive aquatic environmental control measures to prevent ongoing transmission.

*Cryptosporidium*, a protozoan parasite, is an important and widespread cause of enteric infections. Infection is usually spread through contaminated drinking or recreational water or contact with infected animals or persons. A low infectious dose can cause illness, and oocysts can survive for long periods in the environment. (*1*) *Cryptosporidium* is resistant to conventional chlorine disinfection and is a major cause of swimming pool-associated gastroenteritis outbreaks in Australia. (*2*) Risk factors associated with aquatic cryptosporidiosis outbreaks include faecal contamination, inadequate filtration, high bather load and chlorine-resistant oocysts. Currently, no pool water treatment can prevent *Cryptosporidium* contamination. The typical incubation period for cryptosporidiosis is 7 days (range: 1–12 days), and the main symptoms are watery diarrhoea and stomach cramps.

Cryptosporidiosis is a nationally notifiable infectious disease. (*3*) In Victoria, medical practitioners and pathology services are required to notify cryptosporidiosis cases within 5 days of diagnosis. In practice, notification occurs via electronic laboratory notification systems to the Victoria Department of Health. Cases are then allocated to a local public health unit for investigation.

In 2024, Victoria was one of several Australian states to report an increase in the number of cryptosporidiosis cases (38 cases per 100 000 persons, compared with a 10-year average of 14.3 per 100 000), with multiple outbreaks associated with aquatic facilities. (*4*, *5*) While case numbers associated with aquatic facilities were declining by the end of 2024, in early February 2025, the North Eastern Public Health Unit (NEPHU) received notification of a gastroenteritis outbreak linked to a community swimming pool in Melbourne, Victoria. The presence of *Cryptosporidium* was suspected and confirmed on  16 February. This paper describes the rapid investigation of the outbreak, as well as the public health response. This included pre-emptive pool treatment (hyperchlorination) on 14 February.

## Methods

### Setting

The NEPHU covers a mostly urban area with a population of 1.8 million in the northeastern part of metropolitan Melbourne. The pool (Pool A) is a seasonal aquatic facility in the eastern suburbs of the city with three outdoor pools: a 50-m pool with its own filtration system, and a leisure pool and a toddler pool that share the same filtration system. The treated calcium hypochlorite filtration systems were updated in 2024 and are tested every 4 hours while the pool is open.

### Outbreak identification

The outbreak investigation was triggered on 13 February when NEPHU received simultaneous reports of gastroenteritis symptoms among a social group who had swam at Pool A on 4 February and among students at School B who had attended the same pool for a whole-of-school swimming carnival on 5 February. The social group reported their symptoms to the local council on  12 February (Council C, the regulator of the facility), who in turn referred the complaint to NEPHU on 13 February.

The cluster at School B was identified when a gastroenteritis outbreak at a school camp was reported to the neighbouring South Eastern Public Health Unit (SEPHU). SEPHU made the potential link with the school swimming carnival and communicated cases to NEPHU on 13 February.

### Epidemiological investigation

Social group cases and unwell students from School B who had visited Pool A on 4 and 5 February, respectively, were interviewed by telephone using the standard *Cryptosporidium* interview template to ascertain symptom details, symptom onset date and pool attendance. All individuals in the social group (or parents/guardians in the case of minors) were interviewed. Parents or guardians of the symptomatic students were interviewed sequentially based on a list of contact details provided by the school.

Once the outbreak had been confirmed, active case finding was undertaken using an online questionnaire developed specifically for the outbreak (**Supplementary Material**). The questionnaire collected information on pool attendance, symptoms, symptom onset date and school attendance. The questionnaire link was sent to all Pool A patrons, and to parents or guardians of all students at School B, as well as two additional schools that held swimming carnivals at Pool A during 4–13 February. The online questionnaire was anonymous (although an option to leave contact details was provided), giving rise to the possibility of duplicated responses with the telephone interviews. Potential duplicates remained in the analysis.



### Laboratory investigation

Symptomatic individuals were requested to provide a faecal sample for testing. To expediate pathogen identification once the outbreak had been declared (and cryptosporidiosis suspected), Council C staff visited individuals’ homes on 14 February to collect samples. Initial samples were couriered to the Microbiological Diagnostic Unit Public Health Laboratory (MDU PHL) and underwent urgent testing using the FilmArray® Gastrointestinal (GI) Panel (BioFire Diagnostics LLC, Salt Lake City, UT, USA) after hours on 16 February. The GI panel simultaneously detects nucleic acids from bacteria, viruses and parasites commonly associated with gastrointestinal disease.

In addition, collection pots were made available at School B and Council C’s offices, and symptomatic individuals were encouraged to submit a faecal sample for testing. These samples were forwarded to the MDU PHL, with testing for parasites and bacterial GI pathogens performed on the BD MAX System using the BD MAX Enteric Bacterial, Extended Bacterial and Parasite Panels (Becton Dickinson, Franklin Lakes, NJ, USA). All samples were referred to the Victorian Infectious Diseases Reference Laboratory for further viral studies as per state guidelines for the investigation of gastroenteritis. (*6*) Some symptomatic individuals chose to attend a medical practitioner and were tested via alternative pathways. As cryptosporidiosis is a notifiable condition in the State of Victoria, all positive detections were notified to the Victoria Department of Health regardless of where the sample was submitted for testing.

### Outbreak case definitions

A probable case was defined as an individual with onset of gastroenteritis symptoms between 5 and 26 February who had swum in Pool A between 4 and 13 February 2025. A confirmed case was defined as a probable case with laboratory-confirmed *Cryptosporidium*.

The window for symptom onset dates reflects the incubation period for *Cryptosporidium*, and thus spans from 1 day after the first group of unwell individuals swam (5 February) to 12 days post-hyperchlorination of the pool (26 February). The start and end of the risk period, i.e. the dates of potential acquisition in Pool A, were defined by the date the first unwell individual swam (4 February) and the last date before hyperchlorination (13 February).

### Data analysis

Questionnaire data were collected using Microsoft Forms and Microsoft Excel, and descriptive analysis of identified cases (probable and confirmed) was conducted in R (version 4.2.0). An epidemic curve was generated to visualize trends in exposure and symptom onset.

## Results

### Outbreak investigation

In total, our investigation identified 75 cases of cryptosporidiosis: 38 confirmed or probable cases from telephone interviews and 37 probable cases from active case finding (**Table 1**). Faecal samples were received from 19 individuals, of which 16 were positive for *Cryptosporidium* (10 from the social group, 6 from School B). No other pathogens were identified.

**Table 1:** Demographic and clinical characteristics of confirmed and probable cases during a cryptosporidiosis outbreak, Melbourne, Australia, February 2025 (*n* = 75)

Characteristic	Confirmed		Probable		Total	
	*n*	%	*n*	%	*n*	%
**Total**	**16**	**21.3**	**59**	**78.7**	**75**	**100**
**Respondent group**						
**Interviewed student**	**6**	**37.5**	**19**	**32.2**	**25**	**33.3**
**Interviewed pool attendee**	**10**	**62.5**	**3**	**5.1**	**13**	**17.3**
**Questionnaire respondent**	**0**	**0.0**	**37**	**62.7**	**37**	**49.3**
**Sex**						
**Male**	**10**	**62.5**	**8**	**13.7**	**18**	**24.0**
**Female**	**2**	**12.5**	**10**	**16.9**	**12**	**16.0**
**Not stated**	**4**	**25.0**	**41**	**69.5**	**45**	**60.0**
**Age group, years**						
**0–9**	**8**	**50.0**	**1**	**1.7**	**9**	**12.0**
**10–19**	**6**	**37.5**	**32**	**54.2**	**38**	**50.7**
**20–29**	**1**	**6.3**	**0**	**0.0**	**1**	**1.3**
**30–39**	**0**	**0.0**	**4**	**6.8**	**4**	**5.3**
**40–49**	**0**	**0.0**	**10**	**16.9**	**10**	**13.3**
**50–59**	**0**	**0.0**	**3**	**5.1**	**3**	**4.0**
**60–69**	**1**	**6.3**	**6**	**10.2**	**7**	**9.3**
** ≥ 70**	**0**	**0.0**	**1**	**1.7**	**1**	**1.3**
**Not stated**	**0**	**0.0**	**2**	**3.4**	**2**	**2.7**
**Symptoms reported**						
**Diarrhoea**	**9**	**56.3**	**33**	**55.9**	**42**	**56.0**
**Abdominal pain**	**11**	**68.8**	**25**	**42.4**	**36**	**48.0**
**Nausea**	**7**	**43.8**	**27**	**45.8**	**34**	**45.3**
**Headache**	**7**	**43.8**	**18**	**30.5**	**25**	**33.3**
**Vomiting**	**6**	**37.5**	**19**	**32.2**	**25**	**33.3**
**Lethargy**	**3**	**18.8**	**21**	**35.6**	**24**	**32.0**
**Fever**	**2**	**12.5**	**12**	**20.3**	**14**	**18.7**

Telephone enquiries to members of the social group determined that 13 individuals from five families experienced gastroenteritis symptoms with onset dates between 8 and 10 February, 4–6 days after attendance at Pool A on 4 February. Council C confirmed no recent faecal accidents at the pool and no mechanical issues with pool filtration systems.

As previously noted, the cluster of cases among School B students was identified following a gastroenteritis outbreak at a school camp reported to SEPHU. Around one third of students attending the camp (28/80, 35.0%) had gastroenteritis symptoms. In most cases, symptoms were present upon arrival on 11 February, before students had eaten or drunk anything at the camp or had participated in camp activities. Once the potential link to the swimming carnival was made, and NEPHU notified, the investigation focused on identifying symptomatic students who had attended the swimming carnival. It emerged that the swimming carnival had been attended by 426 School B students, and all had swum in the 50-m pool at the aquatic facility. Moreover, approximately 15% (65/470) of all School B students were absent due to gastroenteritis symptoms in the days following the swimming carnival on 5 February. Telephone interviews identified 25 students who experienced symptoms with onset dates between 9 and 14 February (**Table 1**).

A link to the online questionnaire was sent to over 1500 individuals (including 1034 Pool A patrons and 476 parents or guardians of School B students, as well as to parents or guardians of students from two other schools who held swimming carnivals at Pool A on 7 and 12 February), and 197 completed questionnaires were received. Among the respondents, 176 had attended Pool A between 4 and 13 February, of whom 37 (21.0%) experienced symptoms of gastroenteritis. No cases were reported in the two other schools who held swimming carnivals during the risk period.

Demographic characteristics of the 75 identified cases are summarized in **Table 1**. Six individuals identified through active case finding did not provide contact details, and based on their age and school attendance could have also been in the School B cohort who were interviewed by telephone. Among the students who took part in the swimming carnival on 5 February, the attack rate was 6% (25/426); this is likely an underestimate, as not all cases were contacted or interviewed. Among the social group who swam in the pool (*n* = 13), the attack rate was 100%.

**Fig. 1:**
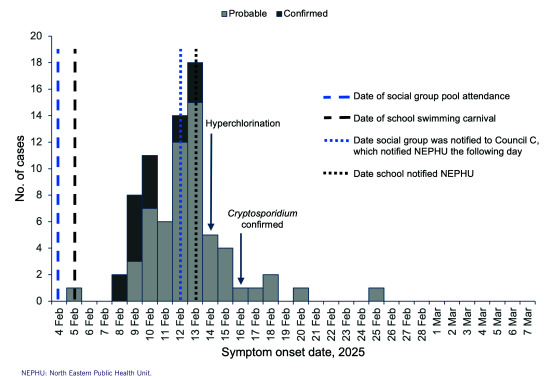
Epidemic curve of cryptosporidiosis outbreak cases by symptom onset date, Melbourne, Australia, February 2025 (^N^ = 75)

Symptom onset dates ranged from 5 to 25 February (**Fig. 1**). The most common symptoms were diarrhoea (56%), abdominal pain (48%) and nausea (45%) (**Table 1**). No cases reported hospitalization. The median time between last attendance at Pool A and symptom onset was 6 days (range: 0–14 days), noting that 18 of the symptomatic online questionnaire respondents reported swimming at the pool more than once during their incubation period. Of those who only attended the pool once, the median time between attendance and symptom onset was 7 days (range: 4–9 days). The median interval between last attendance and symptom onset was similar between the two groups and consistent with the incubation period for C*ryptosporidium* infection. Additionally, the temporal distribution of illness onset was peaked, indicating a point-source exposure, with Pool A being the most likely source (**Fig. 1**).

[**insert** Figure 1]

### Immediate public health response

Given the high attack rates and strong suspicion of cryptosporidiosis, NEPHU recommended on the evening of 13 February that Pool A be closed for hyperchlorination. Council C worked with pool operators to carry out hyperchlorination and a gastroenteritis clean as per state guidelines on 14 February. (*7*-*9*) The pool reopened on 16 February. No water testing was done before hyperchlorination. Water sampling is not part of the recommended cryptosporidiosis outbreak response and was not conducted.

Communications were developed by NEPHU and sent by the school on 14 February to all School B students to inform them of a gastroenteritis outbreak associated with the school swimming carnival, as well as to 1034 Pool A patrons to inform them of an investigation into a gastroenteritis outbreak in people who attended the pool in the first 2 weeks of February. The letter advised symptomatic individuals to be tested or to see their health practitioner to discuss any testing or treatment requirements, to stay at home until 48 hours after symptom resolution and to avoid swimming for 14 days after symptom resolution. The letter also contained general gastroenteritis prevention and healthy swimming advice. (*9*) A poster describing the situation was created and displayed at Pool A.

### Subsequent public health response

*Cryptosporidium* was identified on 16 February, allowing a more focused public health response. The priorities included preventing re-infection of Pool A with *Cryptosporidium* by advising cases to avoid swimming for 14 days after symptom resolution and reminding all pool users of pool hygiene.

An update on the gastroenteritis outbreak was sent to the School B community and Pool A patrons on 18 February, informing that *Cryptosporidium* had been identified as the cause of the outbreak and that the pool had completed treatment. Posters containing the update and healthy swimming messages were displayed at Pool A. Communications were also sent to students at the two schools that had held swimming carnivals at Pool A during the risk period and to schools that were scheduled to hold swimming events in the following week.

NEPHU alerted local general practice clinics to the outbreak and requested clinicians to consider testing for *Cryptosporidium*, especially if there was recent exposure to aquatic facilities and to advise patients with gastroenteritis to avoid swimming for 14 days after symptom resolution. Local pathology services were notified that they may receive an increased number of faecal samples for *Cryptosporidium* testing.

### Surveillance and outbreak closure

Surveillance using the state-wide notification system for laboratory-confirmed *Cryptosporidium* did not identify additional cases linked to the outbreak in the two incubation periods after the last case (24 days), at which time, 7 March, the outbreak was considered closed.

## Discussion

The initial report of a high attack rate of gastroenteritis in two independent groups who swam at Pool A over 2 successive days prompted urgent action at the pool to remove ongoing risk to current pool users. *Cryptosporidium* was the suspected cause of the outbreak due to initial evidence of an incubation period of 4–9 days and the known ability for *Cryptosporidium* to survive standard pool disinfection processes and standard daily cleaning of shared areas.

Hyperchlorination of pools in response to a cryptosporidiosis outbreak is usually only recommended after *Cryptosporidium* is confirmed as the causative agent. However, in this outbreak, the ongoing risk to pool users was considered significant, and immediate pool hyperchlorination was recommended by NEPHU as a precaution. Confirmation of *Cryptosporidium* was received 72 hours after hyperchlorination was performed, suggesting that early hyperchlorination likely prevented further cases. Proactive hyperchlorination has occurred elsewhere, with epidemiological evidence similarly suggesting early control measures prevented a larger outbreak (*10*) and should be considered in similar large outbreaks.

It is unclear if this outbreak would have been identified so quickly without the social group reporting symptomatic individuals to Council C or without the School B camp outbreak. Not all people with gastroenteritis present to a medical practitioner, and fewer still undergo faecal testing. Without early reports, standard surveillance alone may have delayed public health action, increasing the risk of further transmission. This highlights the importance of timely reporting of suspected gastroenteritis outbreaks by the community and schools to local authorities.

The use of e-mail communications and an online questionnaire allowed rapid and large-scale dissemination of information about the outbreak, case management advice and healthy swimming messages, as well as expediting active case finding. The online self-completed questionnaire was a practical and feasible way to assess the size of the outbreak and has since been implemented in other outbreaks at NEPHU.

Questionnaire results indicated continual swimming among symptomatic individuals, which suggests limited knowledge of *Cryptosporidium* in aquatic environments and pool hygiene among pool patrons, as previously reported. (*11*, *12*) While healthy swimming messages were included in all communications to pool patrons and school groups, a wider strategy to raise awareness of healthy swimming and hygiene practices among the general public would be beneficial, especially to reduce the risk of re-contamination of pools, which can occur after hyperchlorination.

### Limitations

The main limitation of this investigation is its reliance on self-reported data and the implicit risk of recall bias among interview and questionnaire respondents. The limitations of active case finding via anonymous online questionnaire included a lack of demographic information; the anonymous nature of the questionnaire resulted in potential duplicated responses with phone interviews. Additionally, it was not possible to distinguish between household transmission (secondary cases) and transmission at the pool (primary cases), a common limitation of outbreak investigations. (*13*, *14*) Lastly, the lack of pre-hyperchlorination water testing precluded confident attribution of *Cryptosporidium* infection to swimming in the pool. Neverthless, epidemiological evidence was strongly suggestive of Pool A as a point-source of the outbreak. However, it should be noted that the detection of *Cryptosporidium* in water does not confirm whether the organism is viable or a risk for human infection, and water sampling is not part of cryptosporidiosis outbreak response in Victoria.

### Conclusion

In conclusion, this outbreak highlights the critical role of rapid outbreak investigation and response, targeted risk communication, active case finding and pre-emptive aquatic facility control measures to prevent ongoing *Cryptosporidium* transmission.
