# Supplementary material for: A large outbreak of cryptosporidiosis at a pool in Melbourne, Australia, 2025: rapid investigation and public health response
Source: Western Pac Surveill Response J. 2026 Jun 30;17(2):1–7. doi: 10.5365/wpsar.2026.17.2.1343 (PMC13403692; doi:10.5365/wpsar.2026.17.2.1343)
Supplement: Supplementary file 1 [file wpsar-17-1343-s001.pdf]

Supplementary Material. **Online questionaire sent to attendees of the swimming pool**

Section 1: Pool attendance

1. Have you attended [pool name] since 4 February 2025?
   a. Yes
   b. No
2. If yes, what dates did you attend?
   a. 04/02/2025
   b. 05/02/2025
   c. 06/02/2025
   d. 07/02/2025
   e. 08/02/2025
   f. 09/02/2025
   g. 10/02/2025
   h. 11/02/2025
   i. 12/02/2025
   j. 13/02/2025
   k. Other: _______________
3. Have you attended any other pool since 4 February 2025?
   a. Yes
   b. No
4. Please specify the pool and dates that you attended.

Section 2: Symptoms

5. Have you experienced any of the following symptoms since 4 February 2025?
   a. Abdominal pain
   b. Diarrhoea
   c. Fever
   d. Headache
   e. Lethargy
   f. Nausea
   g. Vomiting
   h. Other_______________
6. What was your symptom onset date? _________________

Section 3: Demographic details

7. Age (years)____________
8. Do you attend or work at a school?
   a. Yes
   b. No
9. If yes, what school? __________________
10. Would you be happy to be contacted if we require further information?
    a. Yes
    b. No
11. Please insert your contact details (name, phone number, email address).
